# Fatal gangrenous dermatitis with necrotising cellulitis, fasciitis and secondary myonecrosis associated with clostridial organisms in a captive greater rhea (*Rhea americana*)

**DOI:** 10.3389/fvets.2026.1936302

**Published:** 2026-10-12

**Authors:** Lilian Rayanne de Castro Eloy, Markyson Tavares Linhares, Hodias Sousa de Oliveira Filho, João Victor Ferreira Campos, Rodrigo Otávio Silveira Silva, Carlos Augusto de Oliveira Junior, Lucas Rannier Ribeiro Antonino Carvalho, Ricardo Barbosa Lucena

**Affiliations:** 1 Graduate Program in Animal Science (PPGCAn), Universidade Federal da Paraíba, Areia, Paraíba, Brazil; 2 Center for Agricultural Sciences, Universidade Federal da Paraíba, Areia, Paraíba, Brazil; 3 Department of Preventive Veterinary Medicine, School of Veterinary Medicine, Universidade Federal de Minas Gerais, Belo Horizonte, Minas Gerais, Brazil; 4 Department of Physiology and Pharmacology, Karolinska Institutet, Stockholm, Sweden; 5 PatoBiology Laboratory, Graduate Program in Animal Science, Universidade Federal da Paraíba, Areia, Paraíba, Brazil

**Keywords:** *Clostridium chauvoei*, *Clostridium perfringens*, *Clostridium septicum*, infection, ratite, wildlife pathology

## Abstract

Clostridial gangrenous dermatitis is an acute, often fatal, necrotising soft-tissue disease well recognised in commercial poultry, but it is rarely documented in non-poultry avian species and has not been reported as a peer-reviewed case in greater rheas (*Rhea americana*). This report describes a fatal case of gangrenous dermatitis with severe necrotising cellulitis, fasciitis and secondary myonecrosis associated with clostridial organisms in an adult captive female greater rhea. The bird developed acute anorexia, depression, inability to stand, loose stools and a focally extensive, irregular, moderately demarcated lesion in the dorsal pelvic region, characterised by dark-red to black discolouration, multifocal bluish-green areas and feather loss shortly after transport with two other adult rheas, and was submitted for post-mortem examination less than 1 h after death. Grossly, the dorsal pelvic, pericloacal, tail-base and proximal hindlimb regions had extensive necrotising dermatitis, emphysematous cellulitis and fasciitis, and haemorrhagic myonecrosis of the affected skeletal muscle. Histologically, there was severe acute necrotising dermatitis, cellulitis, fasciitis, panniculitis and myonecrosis, with oedema, fibrin, haemorrhage, gas spaces, heterophilic inflammation and numerous bacilli within necrotic subcutaneous and fascial tissues. Impression smears contained numerous rod-shaped bacilli compatible with spore-forming bacteria. Anaerobic culture of affected deep subcutaneous/fascial tissue yielded *Clostridium perfringens* type A, *C. septicum* and *C. chauvoei*, with PCR confirmation performed on bacterial growth/isolates. Histological Gram staining demonstrated Gram-positive rod-shaped bacilli within the necrotic tissue matrix. Aerobic culture and Gram staining of cytological smears were not performed. The findings support a fatal gangrenous dermatitis with necrotising cellulitis, fasciitis and secondary myonecrosis associated with clostridial organisms, although the involvement of aerobic or facultative co-pathogens cannot be completely excluded. This case expands the pathological spectrum of gangrenous soft-tissue disease in ratites and indicates that gangrenous dermatitis with deep cellulitis, fasciitis and secondary myonecrosis should be considered in captive greater rheas with acute emphysematous and necrohaemorrhagic soft-tissue lesions.

## 1 Introduction

Gangrenous dermatitis is recognised in poultry as an acute, often fatal, necrotising clostridial soft-tissue disease involving the skin, subcutis and, in severe cases, the underlying superficial skeletal muscle (1–4). The most directly comparable avian literature includes clostridial dermatitis/cellulitis in commercial turkeys, in which *C. septicum* has been implicated as a primary cause of cellulitis, and both *C. perfringens* and *C. septicum* have been shown to induce cellulitis lesions and mortality (5–7). Although *C. chauvoei* is classically associated with blackleg and clostridial myonecrosis in mammals, it has also been documented in hens, supporting its relevance as a previously reported avian *C. chauvoei* infection (8). The condition is usually associated with *Clostridium septicum* and *Clostridium perfringens* type A, either alone or in combination with other bacteria (2–4). Predisposing factors include skin trauma, immunosuppression, poor sanitary conditions, stress and concurrent disease, all of which may favour anaerobic conditions, bacterial proliferation and toxin production (2–4). Affected birds typically develop an acute clinical course characterised by depression, weakness, reduced feed intake and rapid death, making the disease economically important in commercial poultry production (3, 4).

Gross lesions in avian gangrenous dermatitis commonly include dark-red to bluish cutaneous discolouration, feather loss, subcutaneous oedema, serosanguineous exudation and emphysema, with variable extension into the underlying musculature (1–3). Histologically, reported lesions include oedema, emphysema, haemorrhage, coagulative necrosis and intralesional bacilli in the dermis, subcutis and superficial skeletal muscle (1–4). Therefore, although the term gangrenous dermatitis is traditionally used in avian pathology, the disease may also include prominent cellulitis, panniculitis, fasciitis and myonecrosis when deeper soft tissues are affected (1–4).

This lesion pattern is comparable to other clostridial necrotising soft-tissue infections described in domestic mammals, including malignant oedema, gas gangrene and clostridial myonecrosis (9, 10). In mammals, these infections are commonly associated with *C. septicum*, *C. chauvoei*, *C. novyi* type A, *C. sordellii* and *C. perfringens* type A, and they usually develop in association with tissue injury or other local anaerobic conditions that favour spore germination and toxin-mediated tissue damage (9, 10).

The greater rhea (*Rhea americana*) is the largest South American bird and is maintained in both free-ranging and captive settings (11). Although the literature on diseases of greater rheas remains limited, previous reports have documented infectious, toxic, metabolic and upper respiratory conditions in this species, including avian tuberculosis, viral infections, avian pox, intoxications, rhinitis/sinusitis and serum biochemical alterations in captive or free-ranging birds (12–19). Clostridial enteric diseases have been reported in other ratites, particularly ostriches (*Struthio camelus*) and emus (*Dromaius novaehollandiae*), including enteritis, enterotoxaemia, necrotic enteritis and enterocolitis (20–22). Although the present case was previously documented in the first author’s publicly available master’s dissertation (23), peer-reviewed reports of clostridiosis, particularly clostridial gangrenous dermatitis with involvement of deep soft tissues, are lacking in the greater rhea. Clostridial disease also appears to be rarely documented in other non-poultry avian species, although reported lesions may involve skeletal muscle, intestine and liver, including myonecrosis, enteritis and hepatitis (24).

The present report describes the gross, cytological, histological, microbiological and molecular findings in a fatal case of gangrenous dermatitis with severe necrotising cellulitis, fasciitis and secondary myonecrosis in a captive greater rhea, associated with anaerobic isolation and PCR confirmation of *Clostridium perfringens* type A, *C. septicum* and *C. chauvoei* from affected deep subcutaneous and fascial tissue.

## 2 Case description

An adult female greater rhea (*Rhea americana*) was submitted for post-mortem examination less than 1 h after death. The bird was in moderate body condition, weighed approximately 20 kg and was considered an adult based on body size and weight. Clinical history was limited because of the acute course of disease and incomplete traceability. According to the owner, the bird had no known history of previous illness or veterinary treatment. Shortly before death, it developed anorexia, depression, inability to stand, loose stools and a focally extensive, irregular, moderately demarcated lesion in the dorsal pelvic region, characterised by dark-red to black discolouration, multifocal bluish-green areas and feather loss. The bird had reportedly arrived at noon one day earlier after transport from the state of Rio Grande do Norte, northeastern Brazil, in a small crate together with two other adult rheas. The duration of transport was not recorded. The two remaining rheas remained clinically normal throughout a 1-month follow-up period.

No permanent individual identification or official documentation was available at the time of examination; therefore, the exact origin, age and previous management history of the bird could not be verified. A complete post-mortem examination was performed. Impression smears were prepared from the soft tissue within the lesion and stained with a rapid Romanowsky-type stain. Representative samples of skin, subcutis/fascia, skeletal muscle, lungs, heart, liver, kidneys and intestines were fixed in 10% buffered formalin, processed routinely, embedded in paraffin, sectioned at 4–5 μm and stained with haematoxylin and eosin (H&E). Additional sections of affected subcutaneous/adipose and fascial tissue were stained using a histological Gram method to assess intralesional bacteria. Fresh fragments of affected deep subcutaneous/fascial tissue and skeletal muscle were collected aseptically after reflection of the skin and submitted for bacteriology and molecular analysis.

### 2.1 Gross pathology

The main gross lesion involved the dorsal pelvic region and extended into the proximal hindlimb region, affecting the skin, deep subcutaneous fascia and adjacent superficial skeletal musculature. The affected region was malodorous and crepitant. The overlying skin had a focally extensive, irregular, moderately demarcated featherless and crusted area, characterised by dark-red to black discolouration, multifocal bluish-green areas and multifocal black necrotic foci, consistent with necrotising gangrenous dermatitis (Figure 1A). Multifocal superficial erosions/abrasions, crusting, feather loss and petechial to ecchymotic cutaneous haemorrhages were present at the margins of the lesion, compatible with traumatic disruption of the skin (Figure 1B). Feathers within adjacent apparently normal skin at the margins of the lesion were easily detached. No obvious penetrating wound, deep laceration or puncture tract was identified.

**Figure 1:**
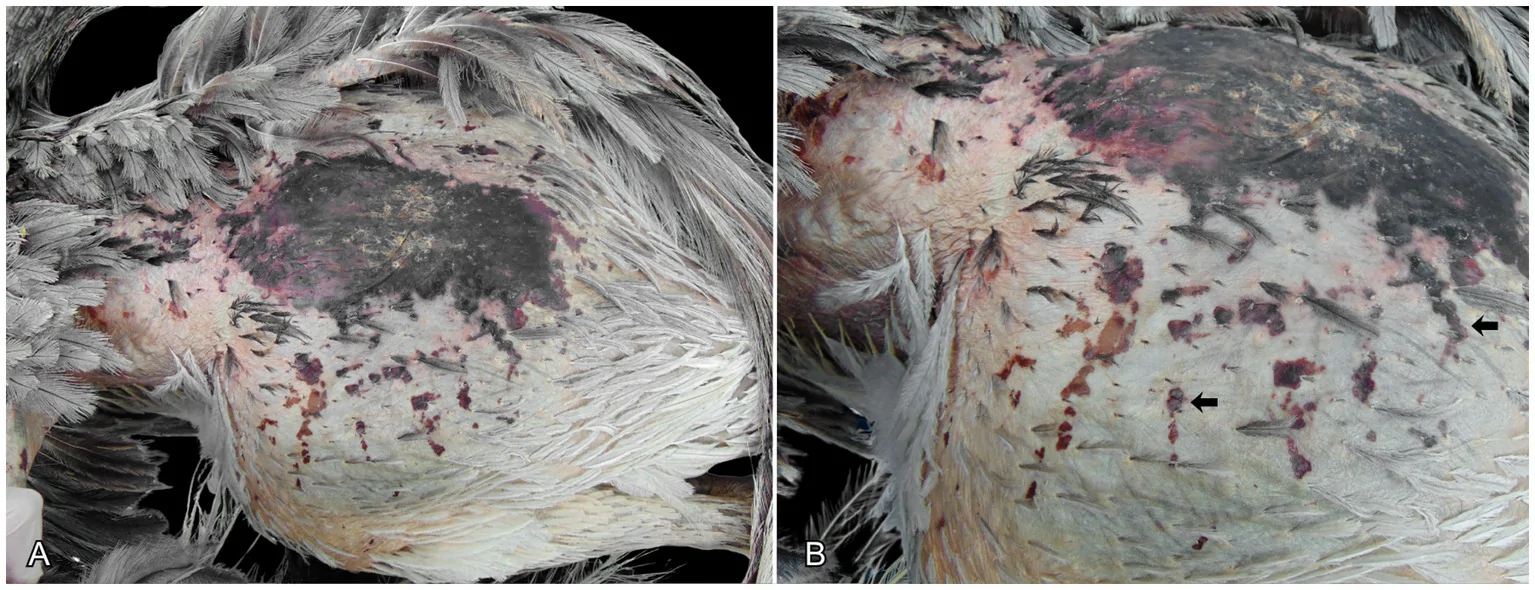
External gross appearance of the cutaneous lesion in a captive greater rhea (*Rhea americana*) with fatal gangrenous dermatitis. **(A)** Dorsal pelvic region showing a focally extensive, irregular, moderately demarcated featherless lesion characterised by dark-red to black discolouration, multifocal bluish-green areas and crusting. **(B)** Higher magnification of the lesion margins, showing multifocal superficial erosions/abrasions, crusting, feather loss and petechial to ecchymotic cutaneous haemorrhages. Arrows indicate representative superficial erosions/abrasions and cutaneous haemorrhages.

After reflection of the skin, the subcutis and fascia of the caudal body wall, pelvic and pericloacal regions, tail-base and proximal hindlimbs were extensively affected (Figure 2A). These tissues were diffusely dark red to black, wet and friable, and were markedly expanded by serosanguineous exudate, malodorous gas-filled cavities and friable necrotic material (Figure 2B). The adjacent superficial pelvic and proximal hindlimb muscles were also extensively involved. On cut surface, these muscles showed loss of the normal muscular architecture, with multifocal to coalescing haemorrhagic and necrotic areas extending through the muscle bundles (Figure 2C). The findings were consistent with severe, extensive, emphysematous necrohaemorrhagic cellulitis, fasciitis and myonecrosis.

**Figure 2:**
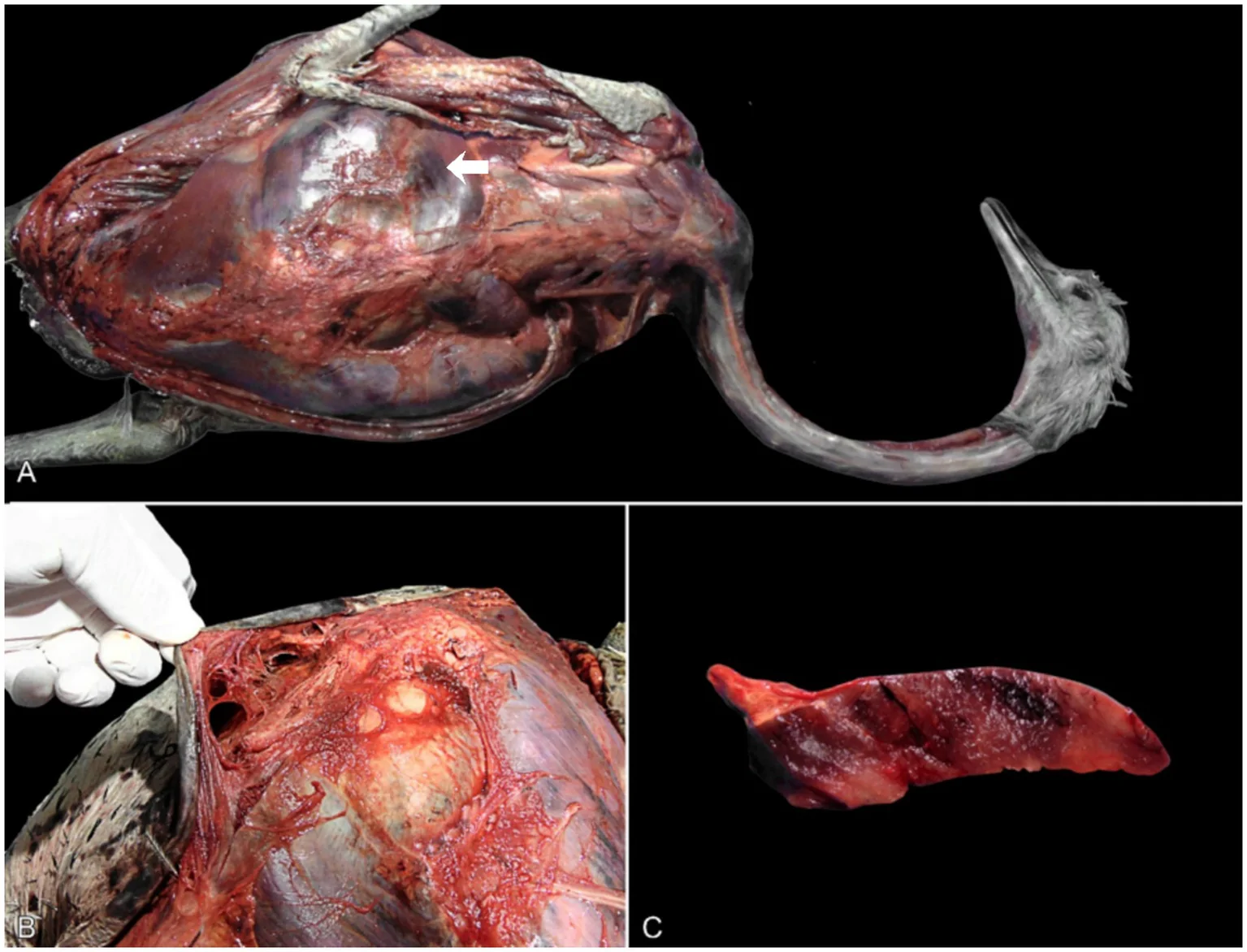
Gross findings of fatal gangrenous dermatitis with necrotising cellulitis, fasciitis and myonecrosis in a captive greater rhea (*Rhea americana*). **(A)** Extensive lesion involving the dorsal pelvic and proximal hindlimb regions, with widespread dark-red to violaceous discolouration and extensive involvement of the subcutaneous tissues, fascia and adjacent superficial skeletal muscles; the arrow indicates a representative dark-red haemorrhagic area within the affected soft tissues. **(B)** Reflection of the skin reveals marked expansion of the deep subcutaneous fascia by serosanguineous exudate, malodorous gas-filled cavities and friable necrotic material, consistent with severe emphysematous cellulitis and fasciitis. **(C)** Section of affected skeletal muscle showing severe dark-red to black discolouration, haemorrhage and myonecrosis.

The lungs were diffusely dark red and wet, with multifocal to coalescing areas of haemorrhage and fluid throughout the pulmonary parenchyma. The ventriculus contained a large amount of compacted green-brown plant material with multiple foreign bodies of variable size, shape and composition, including an irregular ceramic fragment, a dark cylindrical plastic pipe-like foreign body and smaller mineral-like fragments. The intestines contained watery, fetid, greenish-yellow faeces, and the intestinal mucosa had multifocal to coalescing necrotic areas.

### 2.2 Cytology

Impression smears prepared from the cut surface of affected superficial skeletal muscle within the lesion contained numerous predominantly extracellular, rod-shaped bacilli of variable length, arranged singly, in small aggregates and occasionally in short chains. Some bacilli had clear, unstained oval areas compatible with endospores, supporting their interpretation as forms morphologically compatible with spore-forming bacteria. The bacilli were admixed with necrotic debris, erythrocytes and inflammatory cells, composed mainly of degenerate heterophils. Intracytoplasmic bacteria were not a prominent feature.

### 2.3 Histopathology

Sections of skin, subcutis and fascia from the dorsal pelvic region were examined. The superficial epidermis was multifocally effaced and replaced by dense eosinophilic keratin, crusts and cellular debris; remaining epidermal segments showed hypereosinophilia, loss of cellular detail, karyorrhexis and karyolysis, consistent with necrosis. The underlying dermis and subcutis were expanded by marked oedema, fibrin, multifocal haemorrhage and moderate to marked heterophilic inflammation. Deep fascial planes were widely separated by oedema, fibrin, haemorrhage, clear spaces consistent with gas accumulation and necrotic debris (Figure 3A). Because oedema, haemorrhage, necrosis and gas accumulation extensively effaced the subcutis and fascial planes, these structures could not always be sharply separated histologically; however, both subcutaneous and fascial tissues were involved. These findings were consistent with severe, acute, necrotising dermatitis, cellulitis and fasciitis, and panniculitis.

**Figure 3:**
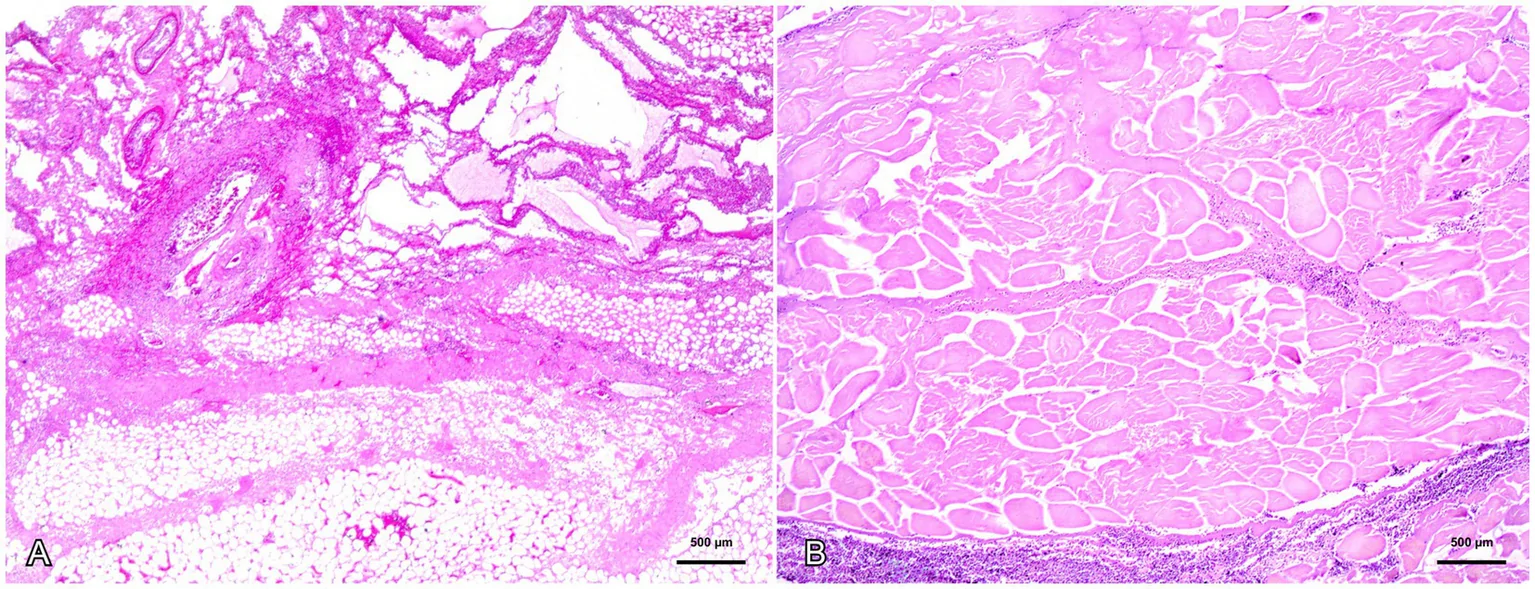
Histopathological findings of fatal gangrenous dermatitis with necrotising cellulitis, fasciitis and myonecrosis in a captive greater rhea (*Rhea americana*). **(A)** Dorsal pelvic integumentary and subcutaneous/fascial tissues showing severe, acute, necrotising dermatitis, cellulitis, fasciitis and panniculitis, with extensive superficial necrosis and marked expansion of the subcutis and fascial planes by oedema, fibrin, haemorrhage, necrotic debris and clear spaces consistent with gas accumulation. **(B)** Adjacent superficial skeletal muscle showing severe acute myonecrosis with haemorrhage and secondary heterophilic inflammation, characterised by hypereosinophilic and fragmented myofibres, loss of cross-striations, segmental myofibre lysis, oedema and haemorrhage. Haematoxylin and eosin stain; bar = 500 μm.

The adjacent superficial skeletal musculature was examined. Myofibres were swollen, hypereosinophilic and fragmented, with loss of cross-striations and multifocal segmental to locally extensive lysis. Endomysial and perimysial spaces were expanded by oedema, haemorrhage and moderate numbers of heterophils, which formed multifocal interstitial aggregates between degenerate and necrotic myofibres (Figure 3B). These findings were consistent with severe acute myonecrosis with secondary heterophilic inflammation.

Within the affected subcutaneous/adipose and fascial tissues, numerous rod-shaped bacilli were identified at high magnification on H&E-stained sections (Figures 4A,B). Bacilli were present both extracellularly within necrotic debris and within macrophages/macrophage-like phagocytic cells (Figure 4B). Histological Gram staining highlighted Gram-positive rod-shaped bacilli within the necrotic tissue matrix (Figure 4B, inset), supporting intralesional involvement by Gram-positive bacilli.

**Figure 4:**
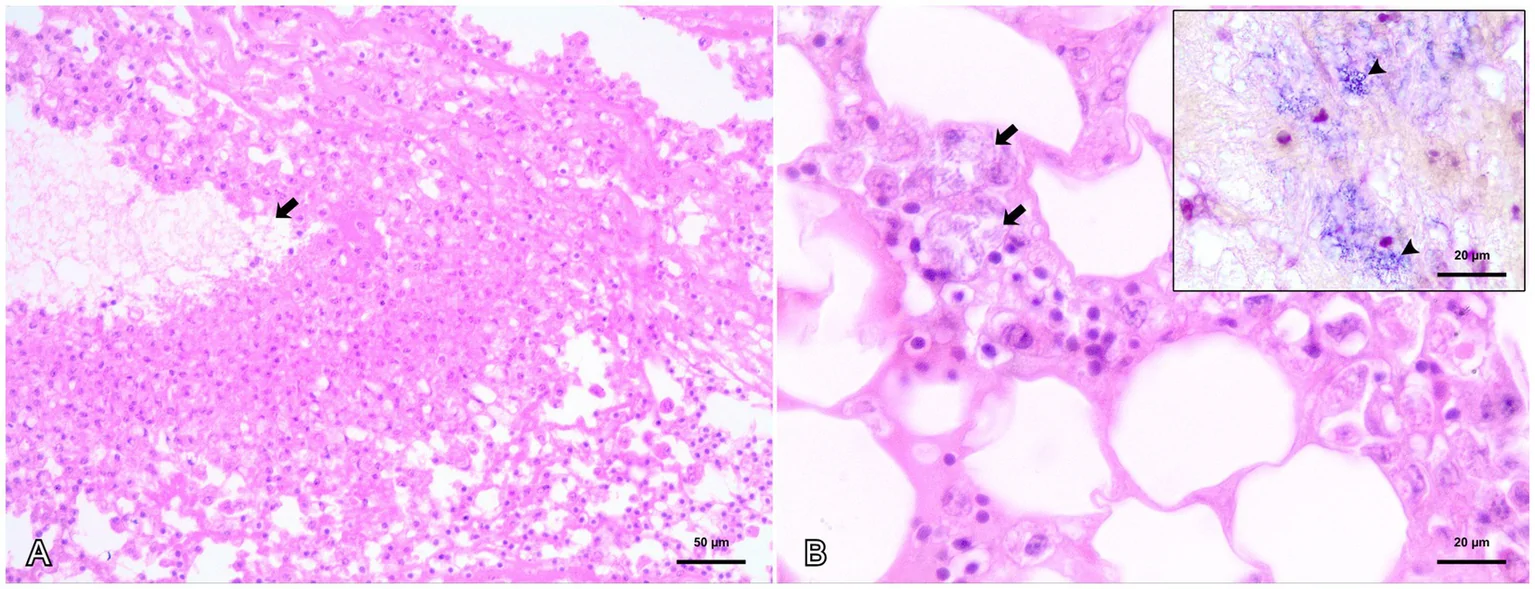
High-magnification histopathological and histochemical demonstration of intralesional bacteria in fatal gangrenous dermatitis associated with clostridial organisms in a captive greater rhea (*Rhea americana*). **(A)** Affected subcutaneous/adipose tissue showing necrotising inflammation, necrotic debris and numerous inflammatory cells; the arrow indicates an area containing bacterial aggregates within necrotic tissue. **(B)** Higher magnification showing macrophages/macrophage-like phagocytic cells containing fine rod-shaped bacillary structures, with additional extracellular bacilli admixed with necrotic debris; arrows indicate representative intralesional bacilli. Inset: histological Gram stain highlighting Gram-positive rod-shaped bacilli within the necrotic tissue matrix; arrowheads indicate representative Gram-positive bacilli. Haematoxylin and eosin stain **(A, B)** and Gram stain (inset); bars = 50 μm **(A)** and 20 μm (**B** and inset).

The lungs showed diffuse oedema and congestion, multifocal haemorrhage and accumulation of eosinophilic proteinaceous material within the respiratory parenchyma. The intestines showed severe villous atrophy, lymphohistiocytic and haemorrhagic expansion of the lamina propria, and multifocal mucosal necrosis with cellular debris, consistent with necrohaemorrhagic enteritis. No relevant histological lesions were observed in the heart, liver or kidneys.

### 2.4 Microbiology and PCR analysis

For anaerobic bacteriology, two fresh samples were collected aseptically after reflection of the skin and processed separately: affected deep subcutaneous/fascial tissue and adjacent affected skeletal muscle. Aerobic culture was not performed. Because this was a routine post-mortem diagnostic case, the microbiological investigation was directed toward anaerobic bacteriology based on the acute clinical course, the malodorous, emphysematous and necrohaemorrhagic gross lesion, and the observation of numerous bacilli in impression smears prepared during necropsy, which raised suspicion of a histotoxic clostridial infection. The samples were plated onto Mueller-Hinton agar supplemented with 5% sheep blood and inoculated into brain heart infusion broth (BHI; Difco, USA). Agar plates and broth cultures were incubated for 96 h under anaerobic conditions. Bacterial growth compatible with clostridia was obtained from the affected deep subcutaneous and fascial tissue. Colonies were subjected to DNA extraction, and multiplex PCR was performed for detection of clostridial species commonly involved in histotoxic clostridial infections (10). *C. perfringens*, *C. septicum* and *C. chauvoei* were isolated from the same affected deep subcutaneous/fascial sample. No clostridial organisms were isolated from the skeletal muscle sample.

For further characterisation, the *C. perfringens* isolate was subjected to toxin-gene PCR targeting the major toxin genes, including the alpha-toxin gene (cpa), beta-toxin gene (cpb), epsilon-toxin gene (etx) and iota-toxin gene (iap/ibp), as well as the beta-2 toxin gene (cpb2), enterotoxin gene (cpe) and NetB-encoding gene (netB) (25, 26). The isolate was genotyped as *C. perfringens* type A based on detection of the alpha-toxin gene (cpa). Aerobic culture was not performed in this case. Therefore, non-clostridial aerobic or facultative co-pathogens could not be assessed microbiologically. However, histological Gram staining demonstrated Gram-positive rod-shaped bacilli within the necrotic tissue matrix, supporting intralesional involvement by Gram-positive bacilli.

## 3 Discussion

This report describes a fatal case of gangrenous dermatitis with necrotising cellulitis, fasciitis and secondary myonecrosis in a captive greater rhea, associated with anaerobic isolation and PCR confirmation of *Clostridium perfringens* type A, *C. septicum* and *C. chauvoei* from affected deep subcutaneous/fascial tissue. The morphology of the lesion closely matched the classic gross and microscopic pattern described for gangrenous dermatitis in poultry, including dark-red to violaceous cutaneous discolouration, necrosis of the overlying skin, serosanguineous subcutaneous oedema, emphysema and involvement of the adjacent superficial musculature (1–4). Histologically, gangrenous dermatitis is characterised by necrosis of the skin and subcutis, accumulation of fluid and erythrocytes within the subcutis, emphysema, intralesional bacilli and necrosis of the underlying skeletal muscle (1, 3). The present case showed the same overall lesion pattern, but in an unusual host species and with particularly extensive involvement of deep subcutaneous fascia and adjacent superficial skeletal muscle. Because aerobic culture was not performed, we interpret this case as fatal gangrenous dermatitis associated with biologically significant clostridial organisms, while acknowledging that involvement of aerobic or facultative co-pathogens cannot be completely excluded. To the authors’ knowledge, this is the first peer-reviewed description of fatal gangrenous dermatitis with necrotising cellulitis, fasciitis and secondary myonecrosis associated with clostridial organisms in a greater rhea.

In the present case, the lesion was centred on the dorsal pelvic region and involved the skin, deep subcutaneous fascia and adjacent superficial musculature. This distribution supports a primary cutaneous/subcutaneous process with extension into deeper soft tissues, compatible with the exogenous or “outside-in” mechanism proposed for gangrenous dermatitis in poultry (2, 3). In this mechanism, disruption of the integument allows clostridial contamination and proliferation within subcutaneous tissues, where local anaerobic conditions favour toxin production and progressive necrosis (2, 3). The crusted and featherless dorsal skin lesion observed in this rhea, together with recent transport and confinement with two other adult rheas, supports local trauma or skin compromise as a plausible predisposing event. However, because the exact transport conditions and previous management history could not be fully verified, the initiating event remains speculative.

The isolation of *C. perfringens* type A and *C. septicum* from affected deep subcutaneous/fascial tissue is consistent with the agents most often implicated in avian gangrenous dermatitis and clostridial dermatitis/cellulitis (1–7). The concomitant isolation of *C. chauvoei* is noteworthy because this organism is classically associated with blackleg and other clostridial myonecrotic syndromes in mammals (9, 10), but has also been documented in hens (8). Its precise pathogenic contribution in this rhea cannot be determined because the lesion was polymicrobial; nevertheless, its isolation from the same deep affected tissue, together with extensive necrohaemorrhagic cellulitis, fasciitis and myonecrosis, supports biological significance rather than incidental detection, particularly because rod-shaped bacilli were demonstrated within the affected tissue matrix on H&E-stained sections and Gram-positive rod-shaped bacilli were highlighted within necrotic tissue by histological Gram staining. In comparative terms, this case broadens the spectrum of clostridial necrotising soft-tissue infections documented outside domestic poultry and reinforces the potential for mixed clostridial infections to produce severe soft-tissue disease in unusual avian hosts (10, 27).

Gangrenous dermatitis and cellulitis in poultry should not be interpreted as exclusively clostridial diseases. In addition to *Clostridium* spp., aerobic or facultative bacteria, including *Staphylococcus aureus*, *Escherichia coli* and *Pseudomonas aeruginosa*, have been reported as potential agents or co-pathogens in avian gangrenous dermatitis and cellulitis (2, 6). In the present case, the diagnostic investigation was focused on anaerobic bacteriology; therefore, the involvement of aerobic or facultative co-pathogens cannot be completely excluded. Nevertheless, the presence of numerous rod-shaped bacilli within necrotic subcutaneous/adipose and fascial tissues on H&E-stained sections, the demonstration of Gram-positive rod-shaped bacilli within the necrotic tissue matrix by histological Gram staining, and the anaerobic isolation of *C. perfringens* type A, *C. septicum* and *C. chauvoei* from affected deep subcutaneous and fascial tissue support the biological significance of the clostridial organisms isolated in this case.

Although the adjacent skeletal muscle showed severe haemorrhage and myonecrosis, clostridial organisms were not isolated from the muscle sample. This finding does not exclude clostridial involvement in the muscular lesion. In gangrenous dermatitis, muscular damage may occur by direct extension from the affected subcutis/fascia and by the local effects of clostridial toxins produced in adjacent necrotic tissues (1–4). In the present case, the strongest microbiological evidence was obtained from the affected deep subcutaneous and fascial tissue, where all three clostridial species were isolated and where numerous bacilli were observed histologically. Therefore, the lesion is best interpreted as fatal gangrenous dermatitis associated with biologically significant clostridial organisms, with severe necrotising cellulitis, fasciitis and secondary involvement of the adjacent superficial musculature.

Post-mortem invasion or contamination by clostridia must always be considered when interpreting microbiological culture results from necropsy material (10). In this case, however, several findings support the biological significance of the isolated organisms. The carcass was submitted for post-mortem examination less than 1 h after death; the gross lesion was severe, extensive, malodorous, emphysematous and necrohaemorrhagic; histological sections showed ante-mortem inflammation, necrosis and numerous bacilli within affected subcutaneous and fascial tissues; and *C. perfringens* type A, *C. septicum* and *C. chauvoei* were isolated from deep affected tissue collected after reflection of the skin. These findings strongly support a true clostridial necrotising soft-tissue infection rather than post-mortem contamination.

Possible predisposing factors in this case included recent transport, confinement in a small crate with two other adult rheas, stress and superficial traumatic compromise of the dorsal skin. These conditions may have favoured disruption of the integument and development of a local anaerobic environment suitable for clostridial proliferation (1–4). Superficial erosions/abrasions and cutaneous haemorrhages were observed at the margins of the lesion, supporting traumatic disruption of the skin as a plausible portal of entry. However, no obvious penetrating wound, deep laceration or puncture tract was identified, and the available information did not allow determination of whether the cutaneous trauma resulted from transport-associated injury, contact with the crate or interaction with other birds. The presence of foreign material in the ventriculus was interpreted as incidental but may indicate suboptimal husbandry or environmental exposure to inappropriate material in captivity. Because the bird lacked permanent individual identification and its previous management history was incomplete, the relative contribution of these factors could not be determined.

The main limitations of this report are related to the absence of ante-mortem clinical evaluation, incomplete traceability, absence of aerobic culture and Gram-stained cytological preparations, and the limited microbiological investigation of non-cutaneous lesions and potential aerobic or facultative co-pathogens. Because the rhea was not examined while alive, detailed clinical data, clinicopathological abnormalities and the exact duration of disease could not be determined. The source of the clostridial organisms also could not be established because the environment, transport crate and remaining rheas were not sampled microbiologically. Although concomitant necrohaemorrhagic enteritis was observed, intestinal tissue was not submitted for anaerobic culture or molecular testing; therefore, the relationship between the intestinal lesion and the clostridial soft-tissue infection could not be determined. In addition, because bacteriology was focused on anaerobic organisms, the involvement of aerobic or facultative co-pathogens could not be completely excluded. Finally, the infection was polymicrobial, and clostridial organisms were isolated from affected deep subcutaneous and fascial tissues but not from skeletal muscle. In addition, although the alpha-toxin gene (*cpa*) was detected in the *C. perfringens* isolate, direct detection of toxins in affected tissues was not performed, and active toxin production by the isolates was not confirmed. Therefore, the relative contribution of each clostridial species to the infection and the precise mechanisms underlying muscular injury could not be determined. Nevertheless, the acute clinical course, prompt post-mortem examination, severe necrohaemorrhagic and emphysematous lesions, numerous intralesional bacilli and anaerobic isolation of three clostridial species from deep affected tissue strongly support a biologically significant clostridial involvement in the necrotising soft-tissue lesion.

## 4 Conclusion

In conclusion, this case indicates that fatal gangrenous dermatitis with necrotising cellulitis, fasciitis and secondary myonecrosis should be included among the differential diagnoses for acute, emphysematous and necrohaemorrhagic cutaneous and deep soft-tissue lesions in captive greater rheas. The isolation of *C. perfringens* type A, *C. septicum* and *C. chauvoei* from affected deep subcutaneous/fascial tissue, together with compatible gross, cytological and histological lesions and histological Gram-stain demonstration of Gram-positive rod-shaped bacilli within the necrotic tissue matrix, supports biologically significant clostridial involvement in this lesion. However, because aerobic culture and direct toxin detection were not performed, the possibility of aerobic or facultative co-pathogens and the precise contribution of each clostridial species could not be determined.

## Data Availability

The original contributions presented in the study are included in the article/Supplementary material, further inquiries can be directed to the corresponding author/s.
